# Digital holographic microscopy: use of artificial intelligence to enable rapid blood culture Gram stain digitization and interpretation

**DOI:** 10.1128/jcm.01627-25

**Published:** 2026-03-30

**Authors:** Kenneth P. Smith, Erin Boulanger, Michael Elkan, Jeffrey Fink, Sofia Cagina, Alena Shamsheyeva, Kyle Spafford, Richard Stahl, Oleg Gusyatin

**Affiliations:** 1 Infectious Disease Diagnostics Laboratory, Children’s Hospital of Philadelphia, Philadelphia, Pennsylvania, USA; 2 Department of Pathology and Laboratory Medicine, Perelman School of Medicine at the University of Pennsylvania, Philadelphia, Pennsylvania, USA; 3 Macula Vision Systems, Tucson, Arizona, USA; Endeavor Health, Evanston, Illinois, USA

**Keywords:** slide scanning, blood culture, Gram stain

## Abstract

Gram staining is one of the most commonly performed tests in the clinical microbiology laboratory. Results are used to both guide empiric antimicrobial therapy as well as downstream laboratory procedures, including culture workup. Despite its ubiquity, Gram staining is time-intensive, operator-dependent, and demands a rapid turnaround time. As such, it is an ideal target for automation. However, currently available automated microscopes struggle to rapidly and accurately collect image data due to physical challenges posed by focusing on micron-sized bacteria on slides with variable stain thickness and intensity. Here, we present the development of digital holographic microscopy, an optical system that scans slides using a multispectral coherent light instead of traditional lenses. This allows for the collection of all image data from a large portion (50%) of a slide. These data are then reconstructed and displayed as full color images. We additionally demonstrate that images collected by this method are qualitatively equivalent to those collected with optical microscopy and can be interpreted by individuals with no previous exposure to automated microscopy.

**IMPORTANCE:**

Gram staining is a commonly performed test that demands significant time and experience. Automation of this task is therefore attractive but has been hindered by physical limitations of traditional optics as currently implemented in automated microscopes. Here, we present the development of digital holographic microscopy, which can collect and display image data without the need for optical focusing. This technology may therefore provide benefit to clinical microbiology laboratories by increasing efficiency of the Gram staining process.

## INTRODUCTION

The Gram stain classifies bacteria into Gram-positive and Gram-negative categories based on differences in cell wall structure. This information is combined with organism shape and spatial orientation to create a complete Gram stain result (e.g., Gram-positive cocci in chain). Gram stains performed directly from clinical specimens provide the first suggestion of whether there is an infection and what the causative organism may be. In clinical practice, these results are used to inform empiric antimicrobial therapy. Staining results are also used within the microbiology laboratory to correlate with culture growth to determine significance of organisms present, and for blood cultures, to select appropriate molecular diagnostic panels.

Despite their ubiquity, Gram stains are one of the most time-intensive tests performed by a clinical microbiology laboratory and require rapid (<60 min) turnaround time for critical specimens (e.g., sterile body fluids, blood cultures). Further, these stains are commonly prepared and read manually, resulting in the need for significant experience to ensure accurate reads. Unfortunately, a shortage of experienced medical laboratory scientists has persisted for the last decade and is likely to continue (1).

To mitigate the impact of the ongoing staffing shortage, researchers have developed methods to improve Gram stain interpretation by use of artificial intelligence (AI) (2–4). However, the development of artificial intelligence models is predicated on the acquisition of large data sets, which functionally requires use of automated microscopy. Although this technology has been widely adopted in anatomic pathology, automated microscopy systems are not commonly found in clinical microbiology laboratories due to size, cost, or the fundamental physical challenges posed by focusing on slides with variable stain thickness using high magnification.

These challenges include data loss due to variability between slides and high focusing failure rates resulting from very narrow depth of focus. Therefore, a large slide area must be sampled to ensure selection of readable fields. Using traditional automated microscopy methods, scanning time is directly related to scanned area, a limitation which may not support rapid turnaround times required clinically. For this reason, some approaches use pre-scanning at lower magnification and acquire only a small subset of the total smear area at high magnification (4). Moreover, interpretation using a stochastic collection of small fields of view has the potential of dismissing pathologically relevant context. For example, current sampling methods consider each field independently and may therefore interpret stain variability across a slide as different Gram reactions rather than as a result of over or under-decolorization.

Here, we report on the development of the MVS 100 instrument, a fully automated digital holographic microscopy system that scans slides using a multispectral coherent light instead of traditional lenses to create holograms containing all data from the sample. Without the need to physically focus on the specimen, data collection is rapid. Images are reconstructed computationally from holograms, which are interference patterns generated by emitted light and light scattered by the specimen on the slide. After image reconstruction, they are subject to AI interpretation and presentation to medical laboratory scientists as full-color images for subsequent confirmation.

## MATERIALS AND METHODS

### Slide preparation and collection

Positive blood cultures from BD BacTec Peds Plus/F pediatric bottles (Becton Dickinson, Sparks, MD) from the Infectious Disease Diagnostics Laboratory at the Children’s Hospital of Philadelphia (CHOP) were used to manually prepare slides. Broth was heat-fixed to a slide at 60°C–70°C until dry and subject to Gram staining (Becton Dickinson, Sparks, MD and Remel, Lenexa, KS). For each positive blood culture, two slides were prepared concurrently. The first slide was stained and read using manual microscopy as part of routine clinical work and was used only for clinical purposes. The second slide was stained but not read or exposed to immersion oil and retained for potential use in this study. Slides were prepared and stained less than 1 h after the time of blood culture positivity across all shifts in our 24 h/day 7 day/week laboratory. All medical laboratory scientists trained on Gram stain reading participated. The number of participants ranged between 22 and 24 individuals throughout the course of the study. Data associating specific scientists and slides were not collected.

Slides were annotated with expected Gram stain reaction and organism morphology based on culture results, after which they were de-identified.

Slides with a single morphology of the most common Gram stain morphologies encountered clinically were considered eligible for the study: Gram-positive cocci in clusters, Gram-positive cocci in pairs and chains, and Gram-negative bacilli. All eligible slides, including repeat positives from the same patient, were included without regard to specimen distribution, thickness, or stain quality.

### Hologram collection

Digital holographic microscopy is based on the principle of light diffraction upon interaction with material on a slide with solid-state photonics used in place of traditional optical lenses. Data are collected using a dedicated instrument, the MVS 100 scanner. Slides are loaded into a sample carrier and scans are initiated via a single click in a user interface controlled by an on-board computer and custom software. Upon initiating a scan, the sample is illuminated with light of various wavelengths using a custom single aperture coherent multi-wavelength light source positioned at a fixed distance from the sample carrier. In this context, coherence refers to light with waves in phase with one another. This coherence is important to ensure that holograms are generated by diffracted light alone and noise is not introduced by out-of-phase (incoherent) waves.

Upon interaction with the specimen, diffracted light is detected by a custom sensor, capturing a set of monochromatic holograms termed interference patterns. Data for each hologram are collected using a single wavelength of light and represents a diffraction pattern derived from the material present on the slide. Hologram collection is accomplished using a standard scanning pattern that remains the same for all slides regardless of specimen location, density, or stain quality. Simultaneously collected area of the slide corresponds to ~100 mm^2^, and total area acquired per sample varied from 600 to 1200 mm^2^ of the central portion of the slide. In total, hologram collection required 4 min per slide. These interference patterns were collected once per slide and are the basis of the analyses described below.

### Hologram quality assessment

Our set of manually prepared slides contained significant variability of specimen distribution and thickness. Therefore, we developed a model to triage holograms for further analysis. We used a vision transformer neural network architecture (5) trained on a set of 545 holograms each covering a 31.9 mm^2^ area of the sample from 75 Gram-stained slides to classify hologram sample areas into one of six categories: no sample present, valid sparse content, valid dense content, invalid (e.g., sample too thick), hardware failure, and undefined debris. Holograms labeled as sparse or dense content are considered valid and selected for image reconstruction. All other results are considered invalid and rejected for further analysis.

### Computational focusing

Vertical distance between the sensor and slide is estimated by creating a z-stack of at least 30 low-resolution hologram propagations spanning approximately 200 µm. For each slice of the z-stack, a non-iterative wave front propagation with a single wavelength (i.e., using a single illumination channel) is used to retrieve the wave front amplitude using the angular spectrum propagation method (6), generating a series of low-resolution grayscale images of each z-stack. Each image is then subject to focus evaluation by contrast assessment (7), and the image with the maximal focus score represents the approximate focal position constituting coarse focus. Computationally robust implementation of this approach takes place on-board the MVS 100 instrument.

### Multi-spectral reconstruction and encoding

The hologram reconstruction algorithm generates a stack of horizontal planes for every hologram acquired at 1-µm steps covering a total of 30 µm around the estimated vertical position of the glass slide surface to capture the full thickness of the sample at any given position on the slide. In contrast to the coarse computational focusing step, the holograms used here are multi-wavelength, that is, constructed from data collected from each of the distinct illumination wavelengths. The holograms are processed using a numerical algorithm that simulates the wavefront propagation from the sample to the sensors. This algorithm iterates between multiple holograms acquired with different illumination wavelengths, recovering the multi-spectral amplitude and phase signals. The MVS 100 on-board implementation is based on the angular spectrum propagation method combined with the iterative Gerchberg-Saxton algorithm (8).

The 3D reconstruction serves as input to an encoding neural network, which transforms the input signal into a compact, low-dimensional representation that captures the sample’s essential features including object morphology and accurate color representations, which are used to precisely determine the focus position for individual objects, such as bacteria and blood cells. In this way, focal planes are defined on a per-object basis rather than per-field or per-slide as would be typical for traditional microscopic methods.

The encoding neural network architecture is based on a deep model (9), enhanced with hybrid convolutional and self-attention components (6). To create the reference data for the training set, 92 slides were imaged with 90 high power fields collected per slide, using an automated version of an Olympus BX43 (Center Valley, PA) conventional microscope with a 60× magnification objective with a numerical aperture of 0.9. Use of higher magnification (e.g., 100× oil immersion) would be impractical due to challenges in automated scanning at this magnification, including unacceptably long scanning time due to a relatively small field of view and high frequency of out of focus fields due to very shallow depth of focus.

Fields were chosen such that they pass the hologram quality criteria outlined above and contain bacteria of reference. By training the network to correlate the holographic data as input with the conventional microscope images as output, the encoding representation of the sample’s relevant features is generated.

### Attention mapping and automated slide interpretation

At the next stage of processing, the encoding representation is provided as input to the interpretation neural network, which identifies morphological features of the sample, including bacterial cells. Reference interpretation categories were assigned based on expected morphology of the organism that grew in culture. The interpretation neural network model is built on the vision transformer architecture using a total sample-based learning approach that requires significantly less manual annotation than approaches that rely on a collection of regions-of-interest. The training set for the identification network consisted of 118 Gram stain slides.

The network generates Gram stain interpretation calls by classifying the sample into the following categories: “Gram-positive cocci in clusters,” “Gram-positive cocci in pairs or chains,” “Gram-negative bacilli,” and “Undefined” for unclassified morphologies. The network then generates attention areas that highlight the bacterial objects important for making the interpretation calls.

### Resulting image reconstruction

The final stage of processing involves generating high-resolution images by combining the multi-spectral amplitude and phase reconstruction with the results from the encoding neural network. Deep model architecture (9) was used that we refer to as the super-resolution neural network fuses these two inputs, resulting in high-resolution color images. The network was trained using the same architecture and reference data as the multi-spectral reconstructing and encoding network. Several images comprising the equivalent of contiguous ~15 high-powered microscopy fields (15–60 total HPFs equivalent per sample, on average) are displayed to the user as supporting evidence for the automated call, including attention map overlay.

A direct comparison between images generated by digital holographic microscopy and standard optical microscopy was performed by collecting data from predetermined positions on slides using digital holographic microscopy and automated optical microscopy (Olympus BX43 using 60× dry objective). The two images were compared side by side, and the same group of organisms was selected manually from each image.

### Automated slide interpretation

An independent validation set of 77 slides not used in model training was used to assess performance of the whole slide Gram stain interpretation model. Each slide was scanned and subject to evaluation by the quality assessment neural network as outlined in the Materials and Methods section. Resulting images were assigned an automated classification using the trained Gram stain interpretation model. Automated interpretations were compared with reference interpretations derived from organism growth in culture.

### Slide interpretation by medical laboratory scientists

A web-based portal was developed to allow evaluation of reconstructed slide images by medical laboratory scientists. Six medical laboratory scientists each classified the same set of 16 slides presented in the portal. These scientists were all proficient in Gram stain reading with tenure in the laboratory ranging from 8 to 30 years but had never interpreted Gram stains from digital images. Prior to accessing the portal, the scientists reviewed a slideshow outlining how to log into and navigate the portal and were shown three representative images of each Gram stain morphology generated by digital holographic microscopy taken from slides that were not part of the 16-slide test set. No additional training or support was given. In the portal, scientists were given the option to classify the slides as one of the three included Gram stain morphologies, or as “indeterminate” if they felt uncertain about the classification. Scientists were not required to review all images and were allowed to decide when they felt they had seen sufficient information to make a call in a manner similar to how slides are read by manual microscopy.

## RESULTS

### Slide collection

**Fig 1:**
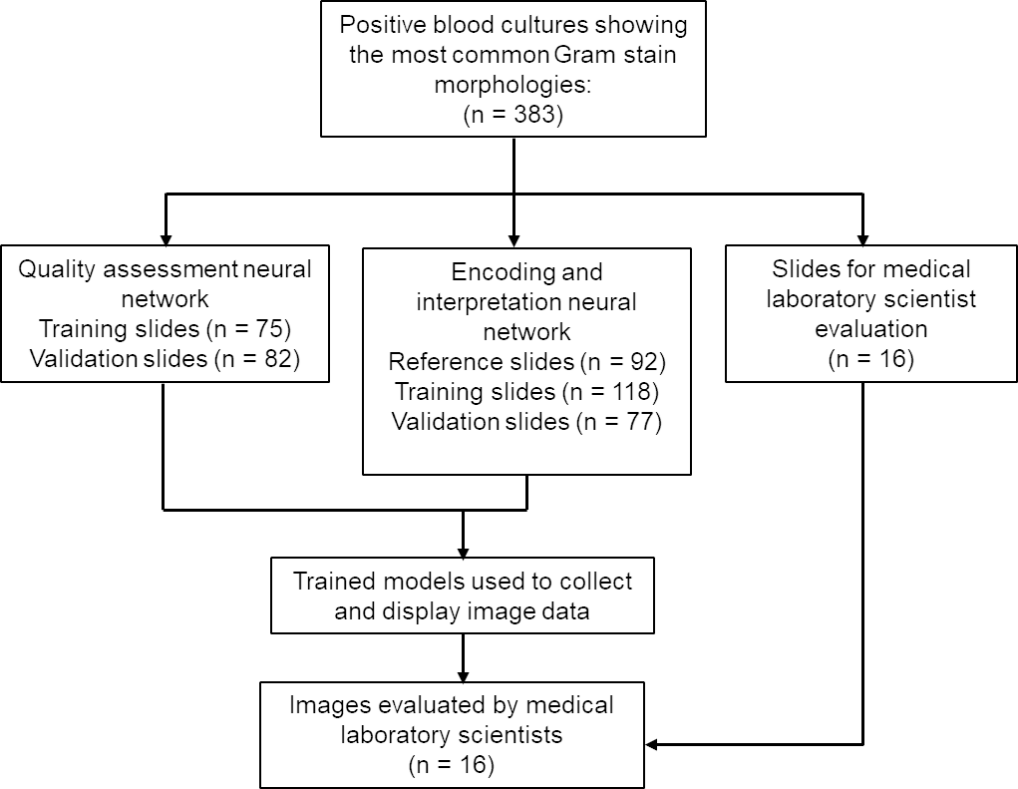
Flow diagram showing slides used in the study.

We collected 383 slides representing the most common Gram stain morphologies (Gram-positive cocci in clusters, Gram-positive cocci in chains, and Gram-negative bacilli). Slide quality, including specimen distribution, thickness, and stain intensity, varied significantly between slides. These slides were used for artificial intelligence model training and evaluation as outlined below, and the flow diagram of all slides used in the study is shown in Fig. 1.

### Hologram collection and quality assessment

Only a subset of slide area represents usable data given variability in slide preparation and staining. When manually examined, uninformative areas of slides are identified and ignored based on a medical laboratory scientist’s experience. Therefore, we developed a quality assessment neural network with the goal of mimicking a scientist’s ability to prioritize fields that contain interpretable data and reject those that do not.

**Fig 2:**
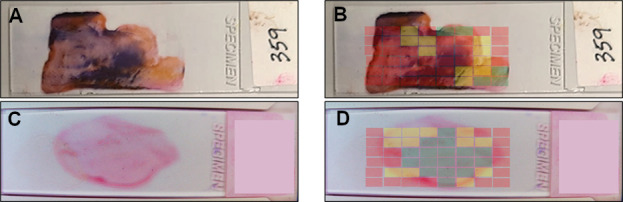
Macroscopic view of example slides (**A and C**) with partially transparent graphical representation of hologram quality assessment data (**B and D**). Green areas represent valid dense content, yellow areas represent valid sparse content, and red areas represent fields that have either no sample present or sample present at unacceptably high density.

Holograms were collected from a total of 532 slides. The sampled region was consistent between slides and included 20 holograms representing 600 mm^2^ or approximately 50% of the total slide area. The quality assessment neural network was trained using a total of 545 holograms collected from 75 slides. Human-classified holograms were used to train a neural network. After training, the network achieved a 99% accuracy of classification as compared with expert interpretation across 82 slides. Per slide, the percentage of valid holograms (sparse or dense content) was, on average, 50% but ranged between 10% and 80%, depending on the quality of the slide. Hologram validity was correlated with macroscopic characteristics, including specimen distribution and thickness (Fig. 2).

### Computational focusing and image reconstruction

**Fig 3:**
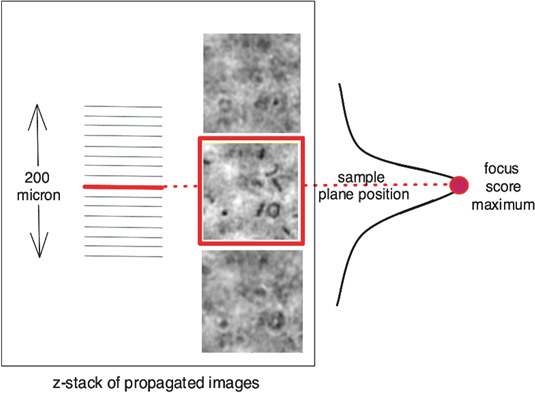
Computational focusing. The position of the glass slide surface within the stack of reconstructed amplitude images is determined as the maximum of the focus score, which is calculated as the image contrast assessment.

In traditional microscopy, precise vertical positioning of an objective is used to achieve optical focus. By contrast, in digital holographic microscopy, the light source remains fixed, and data from the entire thickness of the specimen are collected in a single acquisition. The focal plane is then inferred computationally (Fig. 3).

**Fig 4:**
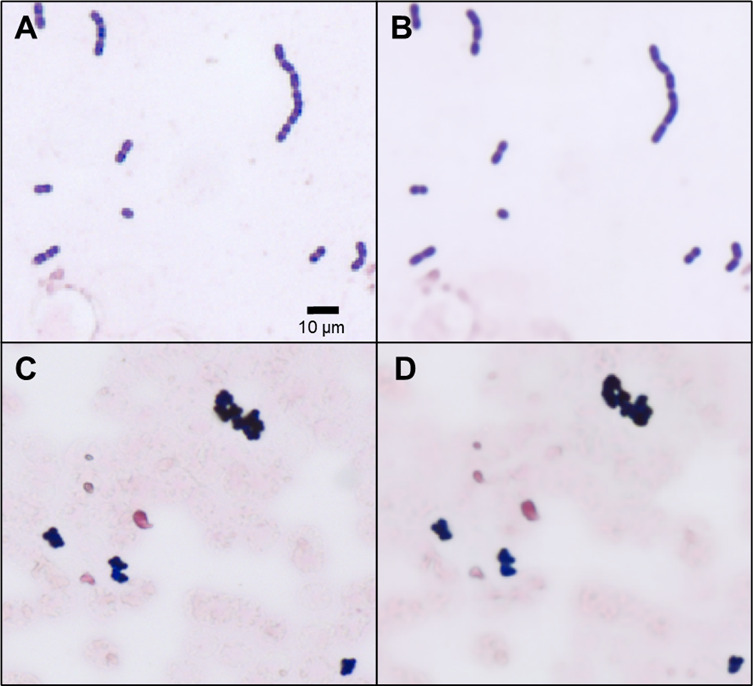
Example images. Images (**A and C**) were collected by optical microscopy. Images (**B and D**) were collected and reconstructed using digital holographic microscopy.

**Fig 5:**
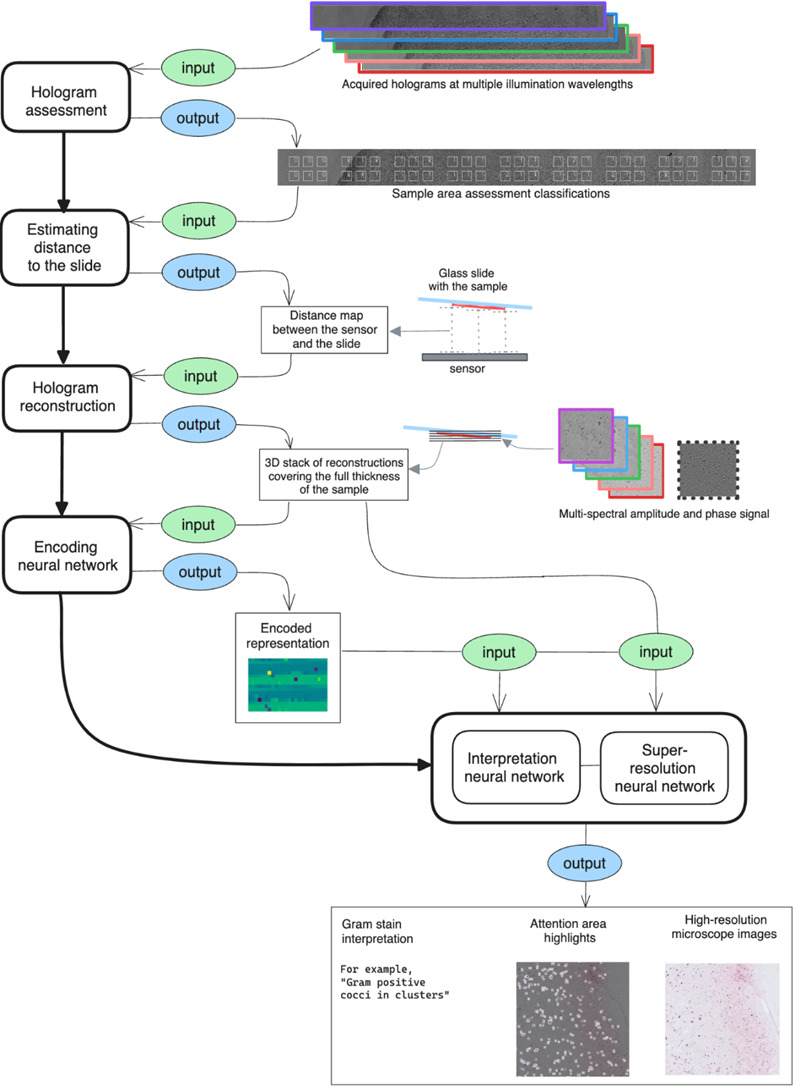
Data collection and image reconstruction workflow.

A subset of holograms with valid quality assessment and computational focusing data were used as input for the image reconstruction neural network. Each reconstructed image represented a field size of 0.7 × 0.7 mm, corresponding to approximately 15 100× oil immersion fields. Resolution was, on average, 125 nm per pixel, qualitatively comparable to the 175 nm per pixel resolution achieved using the 60× dry objective of the Olympus BX43. A direct comparison of both imaging modalities is shown in Fig. 4. The total time from data collection to image reconstruction was 4 min per slide, representing a total area of approximately 600 mm^2^. The complete data collection and image reconstruction workflow is outlined in Fig. 5.

### Automated slide interpretation

Of the 77 slides selected for automated interpretation, 65 were deemed acceptable for further analysis by the quality assessment neural network. Of these, the overall accuracy of the automated interpretation model was 92.3%. Interpretive errors were distributed across categories (Table 1). Accuracy varied by Gram stain morphology and was 93.3% for Gram-negative bacilli, 95.2% for Gram-positive cocci in clusters, and 75% for Gram-positive cocci in chains. When considering only differentiation between Gram-positive and Gram-negative organisms, interpretation accuracy was 93.3% for Gram negatives and 96% for Gram positives.

**TABLE 1:** Confusion matrix for automated slide interpretation

	Automated classification		
True classification	Gram-negative bacilli	Gram-positive cocci in clusters	Gram-positive cocci in pairs and chains
Gram-negative bacilli	14	1	0
Gram-positive cocci in clusters	1	40	1
Gram-positive cocci in pairs and chains	1	1	6

### Slide interpretation by medical laboratory scientists

**Fig 6:**
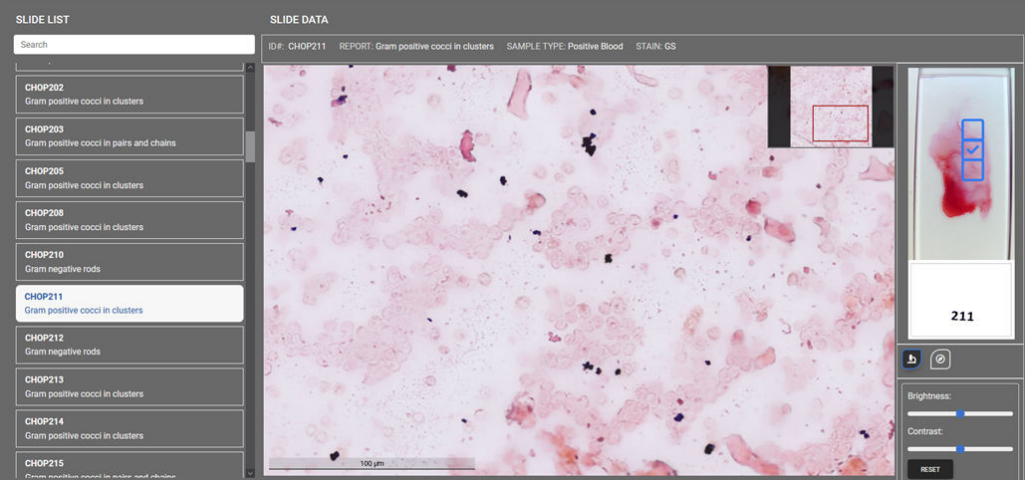
Interactive portal used for slide classification. The portal allows a user to navigate between a slide set (left pane). For each slide, various fields selected by the quality assessment neural network can be accessed (blue boxes on slide image, right pane) and are displayed in the center of the screen. Images can be zoomed and panned using the mouse. Brightness and contrast adjustments are also available. An attention map overlay can be toggled on or off.

Medical laboratory scientists were provided with access to a portal (Fig. 6) containing images generated from a set of 16 slides and instructed to classify them appropriately. For each slide, scientists were able to navigate between images selected by the quality assessment neural network by clicking designated areas on the macroscopic slide image (Fig. 6, blue boxes). For each image, brightness and contrast could be adjusted temporarily but are not saved and do not affect the original file. Pan and zoom capabilities were available using the mouse cursor and mouse wheel, respectively. Non-specific attention mapping data (i.e., a combined attention map that aims to highlight any organism, regardless of Gram reaction or morphology) was able to be overlaid or removed at the scientist’s discretion, but the automated interpretive call was suppressed.

**Fig 7:**
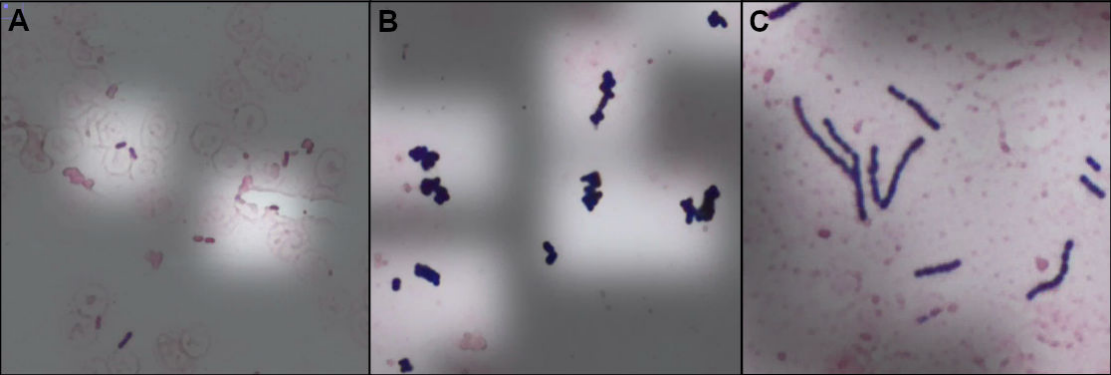
Example images with attention map overlay. (**A**) Gram-negative bacilli. (**B**) Gram-positive cocci in clusters. (**C**) Gram-positive cocci in chains. Images shown are screenshots from images displayed in the portal shown in Fig. 6.

Data were aggregated across all scientists and slides, resulting in 96 Gram stain interpretations (Table 2). Example images with overlaid attention maps are shown in Fig. 7. In total, interpretive calls (i.e., interpretations other than indeterminate) were provided for 91.7% of images. Slides that scientists classified as indeterminate were evenly distributed between Gram stain morphologies. Indeterminate calls were also not obviously operator dependent, with three scientists classifying two slides as indeterminate, two scientists classifying a single slide as indeterminate, and one scientist that did not classify any slides as indeterminate. Further, indeterminate results did not cluster within specific slides, except for a single slide that two scientists classified as indeterminate. Qualitatively, slides called indeterminate showed very sparse distribution of organisms. Where classification calls were made, accuracy was 99%. There was one interpretive discrepancy in which a single scientist interpreted Gram-positive cocci as under-decolorized Gram-negative coccobacilli.

**TABLE 2:** Confusion matrix for medical laboratory scientist interpretation of slide images

	Medical laboratory scientist classification^*b*^			
True classification	Gram-negative rods	Gram-positive cocci in clusters	Gram-positive cocci in pairs and chains	Indeterminate^*a*^
Gram-negative rods	34	0	0	2
Gram-positive cocci in clusters	0	21	0	3
Gram-positive cocci in pairs and chains	1	0	32	3

^*a*^ This category was used when the scientist did not feel confident in deciding the Gram stain reaction and/or morphology of organisms seen.

^*b*^ Data represents aggregated interpretations of six medical laboratory scientists evaluating the same 16-slide dataset.

## DISCUSSION

Persistent staffing shortages challenge the microbiology laboratory’s ability to perform Gram stains accurately within a clinically actionable timeframe (1). In this study, we describe digital holographic microscopy to support this need by rapid digitization of Gram stains. In contrast to traditional microscopy, this method does not require optical focusing and instead reconstructs images computationally from holograms. We employed a full slide encoding foundation model alongside slide level reference data, eliminating the need for tedious image level annotation while retaining robust interpretative ability in line with recent advances in histopathology (10).

We acknowledge several limitations to our study. This work was performed using manually prepared Gram stains, which, to our understanding, reflects the practice of most laboratories. However, automated sample preparation and staining may result in higher consistency between stains. We also used only pediatric blood culture bottles, which yield qualitatively similar stains to adult bottles, but may have subtle differences that impact performance of our models. Further, although our specimens varied in thickness, blood culture broth is a homogenous mixture with abundant organisms distributed relatively evenly across the slide. We identified several situations where sparse organisms resulted in indeterminate calls by medical laboratory scientists, suggesting opportunity for improvement of our quality assessment model.

Polymicrobial blood cultures and cultures containing less common organism morphologies (for example, yeast and Gram-positive bacilli) were not evaluated here due to low frequency at our institution. Although further study of these culture types is needed, pilot studies on polymicrobial specimens suggest that our image reconstruction models can accurately reconstruct images of multiple bacterial morphologies simultaneously as well as yeast (data not shown). Other specimen types evaluated in the microbiology laboratory (for example respiratory or wound specimens) present substantially higher variability in organism distribution compared to blood cultures. The performance of this model on such specimen types was not investigated here, and additional work is warranted to determine whether our method of data collection and interpretation is generalizable to other laboratories, blood culture bottles, and specimen types.

In addition to expanding our Gram stain models to other modes of preparation, we also see promise in this technology for other specimen types. Specifically, hologram collection necessarily collects data comprising the entire three-dimensional structure of all objects on a slide, allowing computational reconstruction of large z-stacks without need for optical focusing on each plane. As such, in future work, we aim to expand the use of digital holographic microscopy to very thick specimens, including wet mount parasitology slides.
